# Gelsemium

**Published:** 1889-08

**Authors:** A. McNeil

**Affiliations:** San Francisco


### GELSEMIUM.

A. McNeil, M. D., San Francisco.

Permit me to warn you against the delusion that this drug is a specific for certain fevers, or that it is a substitute for Aconite in malarious regions. Specifics and substitutes are the refuge of idlers, and are now costing more lives than alcohol.

The pivotal symptom of Gelsemium is relaxation, both mental and physical. True, sometimes it is indicated by symptoms which correspond to the secondary effects of the drug; but these are the exception. We see this relaxation manifested in a desire to be alone, and he is irritable and sensitive if disturbed. And in a great lack of courage, both mental and physical: he is afraid to appear in public, as to speak or sing. If he attempts to do any of those things there is a relaxation of the muscles, as of the eyelids, so that they fall down, or the limbs refuse to obey the will; the womb loosens its hold on its immature contents; the sphincters lose their contractility, so that diarrhœa ensues. For these reasons he desires to be quiet, does not wish to speak nor to have any one near. This fear is more like that of Aconite than of any other drug. But there is a distinction in that the Aconite fear is caused by a morbid excitability, while with the fear of the yellow jasmine is that of this general relaxation that deprives him of courage and energy. By keeping this in mind you will be able to differentiate in threatened abortion and premature labor. Opium has a resemblance also in meeting the bad effects of fright, but its effects are deeper and more profound, as seen in the unconsciousness, convulsions, twitching around the mouth, hot, red face, and the fæces pass involuntarily.

The physical relaxation is revealed by the muscles refusing to obey the will, the eyelids droop.

To follow out the affections which Gelsemium cures on the different organs will now demand our attention.

In the sensorium we find dizziness and blurring of vision, gradually increasing, all objects appear very indistinct, vertigo, spreading from occiput over whole head (Silicium has vertigo rising from the neck into the head, but it is attended with nausea); the pupils are dilated, and there is dim sight and general depression; this condition may arise from the heat of summer. The child is dizzy when carried, seizes hold of nurse, fearing that it will fall. This must be differentiated from a similar symptom of Borax and Cuprum; with Borax the child is afraid of falling only when it is being lowered or when its legs are elevated, as in putting on its diaper. With Cuprum the child is afraid of every one who approaches him, so that it is the fear of being separated from its nurse which actuates it to cling.

The head symptoms of Gels. deserve careful attention, as not only does it cure headaches, but in fevers these symptoms may be of great value in making up a picture of the case. It cures headache in which the patient gets blind before the headache; with Kali-bich. the blindness comes first, and as the pain increases in intensity the dimness of sight decreases; is relieved by sitting, and by reclining the head and shoulders upon a high pillow, by profuse micturition, similar to Ignatia, Kalmia, and Silicea; and by shaking the head. It also cures a headache which begins in the neck and extends over the head, causing a bursting sensation in forehead and eyeballs; with Sanguinaria, it begins in the occiput, spreads upward, and settles over the right eye; with Silicea it ascends from the nape of the neck to the vertex, and then over both eyes or to the eyeballs, especially the right one.

Gelsemium is the remedy when there is a sensation of a band around the head above the ears; with the Bromide of Ammonia there is the same feeling, only it presses hardest just above the ears.

I have already mentioned the drooping of the eyelids from the weakness of the muscles. Causticum has the same condition. Rhus-tox. has a heaviness and stiffness of the lids, making it difficult to move them. Gelsemium has a smoky appearance before the eyes, with a pain above them. It has frequently cured amaurosis caused by masturbation, with the charateristic relaxation of mind and body. In the febrile and other conditions diplopia may occur. This is one of the remedies for that condition, but it lacks the severity of the double vision of Bell. and Stramonium, as with this drug it occurs only when inclining the head toward the shoulders or on looking sideways, and is controllable by an effort of the will.

There is one symptom of the tongue which, in fevers, may be of decisive importance, viz.: can hardly put tongue out it trembles so, as in Lachesis.

Gelsemium will cure that diarrhœa which is caused by sudden depressing emotions—fright, grief, bad news, excitement, or fear of any ordeal, such as appearing in public, a surgical operation, going into battle, etc. Ignatia and Opium are to be remembered in these cases, but by remembering the concomitants no difficulty will be experienced in making the right choice. This same emotional cause may produce involuntary discharge of urine. I have already mentioned that in headaches relief is produced by frequent emissions of copious, clear urine.

Seminal weakness, with the relaxed condition of mind and body mentioned above, requires Gelsemium. It will be necessary to bear Phosphoric acid in mind in these cases, but when the latter drug is indicated, there is the same relaxation of the mind as with the former remedy, but it is more profound, amounting to apathy and indifference, and accompanied by sleepiness. This drug has been recommended as a specifie for gonorrhœa, but beware of specifics, as they will disappoint you in nineteen cases out of twenty.

In diseases of women, you must remember this drug in threatened abortion from sudden depressing emotions. You must observe the difference between this and Aconite, which is very striking, and Opium, which has stupor, while this only has mental and physical relaxation. In labor, the jasmine has a well-deserved reputation. It may enable you to dispense with the forceps when the pains go through to back, and then upward, causing the child to ascend instead of descend. Chamomilla has long-continued pains, which *shoot* upward. Perceive the difference in the character of the pains, although the direction is the same, and remember the ill-humor of the Chamomilla patient. In rigidity of the os, keep Gelsemium in mind. Belladonna is often indicated in this condition, but you will have no difficulty in differentiating from the concomitants. The labor may also be impeded by a wave-like sensation from the uterus to the throat, ending with a choking feeling. This may be a premonition of convulsions, and the Gels. if given, will avert the danger. Frequently labor may be delayed by that mental and physical relaxation so characteristic of this drug: the pains have stopped, os dilated, and she is listless.

In heart disease Gelsemium will cure if he fears that unless he moves constantly his heart will stop beating. With Digitalis the direct opposite prevails ; he fears to move for fear his heart will stop beating. The pulse of Gelsemium offers valuable information. It is full and slow (Opium also), frequent, soft, and weak, so as to be almost imperceptible.

He may suffer from pain in the neck, and under the left shoulder-blade, or will feel a dull aching in the lumbar and sacral regions, with the characteristic inability to control his muscles.

In all or any of his limbs he may be unable to control them by an effort of the will. And he may from this relaxation have great fatigue in the legs after slight exercise.

It is in fevers that Gelsemium has won the most honor. In intermittents the chill begins in the hands and feet, similarly to Natrum-mur. In nervous chill the patient wants to be held, so does the Lachesis patient, but with Gelsemium the skin remains warm. In fever, without thirst, he wants to lie still and rest; this is because he is so relaxed mentally and physically that movement is too much of an exertion for him; with Bryonia it is because movement causes pain. Sometimes we see an intermittent in which the chill is especially along the spine, running up the back from the loins to the nape of the neck in waves following each other rapidly. Gels. is the remedy.

In all the complaints which Gelsemium cures, alcoholic stimulants relieve.

